# Development of a Sustainable Analytical Method Using High-Performance Liquid Chromatography (HPLC) and Dissolution Studies for Quality Control of Ibuprofen Soft Gelatin Capsules from Brazil

**DOI:** 10.1021/acsomega.5c13413

**Published:** 2026-06-23

**Authors:** Laura Beatriz Souza e Souza, Ana Carolina Ferreira Nunes, Ivana Ferreira Simões, Thaís Luz de Souza, Aníbal de Freitas Santos Júnior

**Affiliations:** † Department of Life Sciences, 74363 Universidade do Estado da Bahia, Silveira Martins, 2555, Cabula, Salvador 41150-000, Bahia, Brazil; ‡ Agricultural Technology Center of the State of Bahia, Av. Milton Santos, 967, Ondina, Salvador 40170-110, Bahia, Brazil

## Abstract

Ibuprofen is one of the first anti-inflammatory drugs, and it has recently been developed in soft gelatin capsule form. Therefore, it is the only oral pharmaceutical for which there is no defined quality control method by pharmacopoeias. This study aims to develop an innovative and sustainable HPLC-DAD method for the determination of ibuprofen in soft gelatin capsules and to compare dissolution profiles and kinetics. The optimal conditions for the chromatographic method were a mobile phase (isocratic) consisting of 70% ethanol and 30% of 0.5% acetic acid at a flow rate of 0.8 mL min^–1^, a column temperature of 35 °C, a detector set to 220 nm, and a run time of 5 min. The metrics for assessing the method’s sustainability were calculated based on the National Environmental Methods Index (NEMI) and the Analytical Greenness Metric (AGREE), yielding results of 2/4 and 0.72, respectively. Validation showed LOD and LOQ results of 0.7768 and 1.3673 mg L^–1^, respectively. Precision had a deviation below 5% and a working range of 1.37 to 20 mg L^–1^. No matrix effect was identified, and the method proved to be robust. The optimal dissolution conditions were defined through experimental planning as follows: 700 mL of phosphate buffer (pH = 7.2), 75 rpm, apparatus 2 (paddle), and 60 min of testing. The ibuprofen release percentage in the capsules ranged from 81.96 to 105.1%. Dissolution efficiency ranged from 32.17 to 45.97%. All samples exhibited first-order kinetics, with only three samples showing an *F*2 value above 50. No Brazilian studies were found that determined the presence of ibuprofen in soft gelatin capsules using high-performance liquid chromatography (HPLC), multivariate analyses, or expanded kinetic studies. Therefore, the developed method is innovative and more sustainable than others described in the literature according to the sustainability parameters. The method was validated for accuracy, LOD, LOQ, matrix effect, and robustness, ensuring effectiveness.

## Graphical abstract

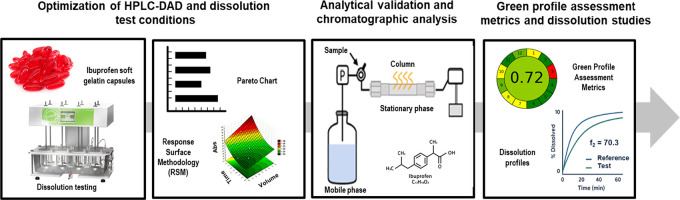

## 1 Introduction

In the 1960s, Stewart Adams was tasked with developing a new painkiller for rheumatoid arthritis that had fewer side effects than commonly used drugs such as paracetamol (acetaminophen), corticosteroids, and acetylsalicylic acid (ASA). After synthesizing over 200 compounds, the first successful phenylpropionic acid derivative was discovered, and a patent was filed for 2-(−4-isobutylphenyl)­propionic acid (Figure 1).^1,2^

**Figure 1:**
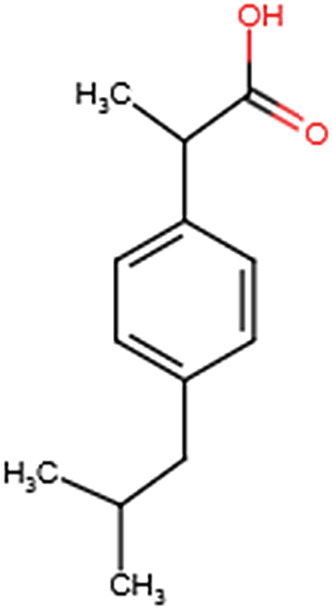
Chemical structure of Ibuprofen.

Ibuprofen was approved for sale with a prescription in the United Kingdom in 1969, the same year it was registered in Brazil. However, due to its safety and tolerability profile, the drug was permitted to be sold over-the-counter in the 1980s.^2^ The increased use of this drug has made it more important to ensure its quality and safety in the different pharmaceutical formulations.

In Brazil, ibuprofen is marketed in various concentrations in the form of tablets, suspensions, and soft gelatin capsules.^3^ The soft capsule is the only form for which no methodology is available in the pharmacopoeias.^4,5^ Pharmacopoeias determine the tests that must be performed on the active pharmaceutical ingredient and its forms. In the absence of information in compendia, industries must adopt other official pharmacopoeias or develop and validate methods capable of identifying and quantifying drugs in various pharmaceutical forms and their active ingredients.^6–8^

Therefore, developing innovative methodologies for drug quality control testing is a significant contribution to research, as it helps develop efficient methods, reduce costs and time, and increase the industry’s analytical frequency. Additionally, analytical methods must align with green chemistry principles to eliminate or reduce the use of environmentally harmful solvents and minimize pollutant emissions, thereby promoting sustainability.^9^ In this context, techniques such as high-performance liquid chromatography (HPLC), especially when associated with chemometric tools, have become widely used in drug analysis due to their advantages, including reduced solvent consumption and improved analytical efficiency.^10–12^

A dissolution test provides an analysis of the process only after it ends. Therefore, a multipoint analysis is necessary to obtain details about drug release through dissolution profiles.^7^ In the pharmaceutical industry, dissolution profiles are used for formulation development and optimization, as well as for assessing drug quality and stability and evaluating changes to formulations and manufacturing processes.^13,14,10^

Eraga et al.^15^ determined the ibuprofen content in tablets using ultraviolet (UV) and high-performance liquid chromatography (HPLC) methods. The authors used acetonitrile, a toxic and environmentally unfriendly solvent, as well as a buffer with a mobile phase. Our study aims to adhere to sustainability principles and use green-chemistry-compliant solvents to determine the ibuprofen content of soft gelatin capsules. Additionally, no Brazilian studies were found that determined the presence of ibuprofen in capsules using HPLC, multivariate analyses, or expanded kinetic studies (e.g., dissolution efficiency and kinetic models). Therefore, this study aims to develop an innovative and sustainable HPLC-DAD method that compares profiles and studies the dissolution kinetics. This method will contribute to the quality assessment of soft gelatin capsules containing 400 mg of ibuprofen that are marketed in Bahia, Brazil.

## 2 Materials and Methods

### 2.1 Materials, Reagents, and Samples

The reagents used for the HPLC-DAD determination were ethanol and acetic acid (HPLC grade) and ethyl ether (PA grade). Ibuprofen analytical standards (Sigma-Aldrich, St. Louis, MO, USA) were used to prepare the analytical curves. The following substances were used to prepare the solutions for the dissolution method: sodium hydroxide (NaOH) and monopotassium phosphate (KH_2_PO_4_). Water obtained from an ultrapure Milli-Q system (18.2 MΩ cm^–1^; Repligen Bioscience, Argentina) was used to prepare solutions and wash materials.

The reference chemical substance (RCS) of ibuprofen was purchased from the National Institute for Quality Control in Health, Oswaldo Cruz Foundation (Fiocruz, Brazil). Samples of soft gelatin capsules containing 400 mg of ibuprofen (reference, similar, and generic) were obtained from drugstores in the state of Bahia, Brazil. The samples were named R03, R69, R96, S31, S37, S94, G36, G61, and G78. The ibuprofen soft gelatin capsules were subjected to average weight and disintegration tests following official compendia. The Brazilian and American pharmacopoeias^4,5^ does not have a method for the dissolution of ibuprofen soft capsules.

### 2.2 Instruments

The average weight was determined using an analytical balance with a precision of 0.001 g (±0.0001 g) (model ATX224R, Shimadzu, Kyoto, Japan). For the disintegration test, a disintegrator (model 301/AC 01, Nova Ética, São Paulo, Brazil) was used. The dissolution test was performed using a dissolver (model 708-DS, Agilent Technologies, Santa Clara, CA, USA) with *n* = 6 tests. A pH meter (Gehaka, model PG2000, São Paulo, Brazil) was used for pH adjustment. A high-performance ultrasonic bath (model P60 H, Elma, São Paulo, Brazil) operating at 37 kHz was used. A high-performance liquid chromatograph (model 1260 Infinity II, Agilent Technologies, Santa Clara, CA, USA) was used with a quaternary pump operating at 600 bar, an automatic autosampler, a Zorbax Eclipse Plus C18 reverse-phase column, and a diode-array detector (DAD). All grams, retention times, and areas were acquired from OpenLab CDS II software.

### 2.3 Calibration Standards

A reference stock solution of ibuprofen (100 mg L^–1^) was prepared with RCS in ethanol. Calibration standards ranging from 1 to 20 mg L^–1^ were prepared from the stock solution by appropriate dilution. The standards were stored and analyzed in triplicate by HPLC-DAD at 220 nm.

### 2.4 General Tests (Weight Variation and Disintegration Tests)

All physical assays (weight uniformity, average weight, and disintegration) were performed according to the Brazilian Pharmacopoeia.^7^ Each capsule was emptied, cleaned, and weighed again. The weight difference between the filled and empty capsules determined the weight. Then, the average weights, standard deviation, and individual deviation of each capsule were determined in relation to the average. Brazil^4^ establishes a maximum variation of ± 7.5%, with no more than two units tolerated outside the specified limits in relation to the average weight; however, none can exceed or fall below twice the indicated percentages.

Six units (soft gelatin capsules) of each sample were tested in the disintegration test apparatus under the following conditions: distilled water as the disintegration medium at 37 ± 1 °C for 30 min. Each capsule was placed in a tube in the basket, and then a disk was added to each tube. Finally, the equipment was activated. After the specified time elapsed, the contents of each tube were observed. All tablets must be fully disintegrated.^4^

### 2.5 Factorial Experimental Design and Optimization of Dissolution Test Conditions

The dissolution test determines the amount of the active substance that has dissolved in the dissolution medium. For tablets and soft capsules with ibuprofen, the dissolution medium specified in the monograph and in some articles was phosphate buffer at pH 7.2, which was used in the dissolution vessel at 37 ± 0.5 °C with apparatus 2 (paddle).^4,5,15^ At predetermined time intervals, an aliquot was removed and filtered before analysis. The medium was replaced with the same heated medium at 37 °C. The sample was quantified using the method developed and validated in this study.

Neither the Brazilian nor the American pharmacopoeias^4,5^ have a method for dissolving ibuprofen soft gelatin capsules. To evaluate the influence of the variables in the dissolution test and find the optimal conditions, a factorial experimental design with three variables (2^3^) and a triplicate of the central point was performed. Thus, 11 experiments were generated (Table 1). The analyzed variables were volume (mL), rotation speed (rpm), and time (minutes). The temperature (37 ± 0.5 °C) and apparatus (paddle) were maintained for all dissolutions according to the Brazilian and American pharmacopoeias and Brazilian guidelines.^4,5,16^

**Table 1:** Experimental Domain of Factorial Experimental Design 2^3^ to Define Dissolution Conditions

levels	variables		
	rpm	volume (mL)	time (min)
1	100	900	60
0	75	700	45
–1	50	500	30

factorial experimental design (2^3^)			
experiment	rpm	volume (mL)	time (min)
1	–1 (50)	–1 (500)	–1 (30)
2	+1 (100)	–1 (500)	–1 (30)
3	–1 (50)	+1 (900)	–1 (30)
4	+1 (100)	+1 (900)	–1 (30)
5	–1 (50)	–1 (500)	+1 (60)
6	+1 (100)	–1 (500)	+1 (60)
7	–1 (50)	+1 (900)	+1 (60)
8	+1 (100)	+1 (900)	+1 (60)
Central point	0 (75)	0 (700)	0 (45)
Central point	0 (75)	0 (700)	0 (45)
Central point	0 (75)	0 (700)	0 (45)

The responses (peak area) were analyzed using ANOVA, and a Pareto chart was plotted with Statistica 7.0 software. This allowed us to investigate the effects and interactions of the variables in the dissolution tests.

### 2.6 Dissolution Studies

Dissolution was tested in a six-tank dissolver at a medium temperature of 37 ± 0.5 °C using apparatus 2 (paddle). The other conditions were defined based on a factorial experimental design, Pareto chart analysis, and the application of the Doehlert matrix after optimization. To obtain the dissolution profile, 10 mL samples were withdrawn at predetermined time intervals (0, 5, 10, 15, 20, 30, 45, and 60 min) and replaced with an equal volume of fresh medium to keep the total volume constant. The collected samples were filtered using a 0.45 μm membrane filter for HPLC-DAD analysis at 220 nm. The peak area was converted to concentration using the equation from the analytical curve. Calculations were performed considering the amount of drug removed in each aliquot, and the results were expressed as a percentage over time.

Dissolution efficiency (DE), a parameter used to evaluate the equivalence of pharmaceutical products, was calculated using the trapezoidal rule to obtain the dissolution profiles.^17^ This parameter was obtained from the area under the drug dissolution curve in relation to time (*t*) in minutes (AUC_0‑*t*_), corresponding to 100% of the label value (AUC_TR_). DE was expressed as a percentage and can be defined by the following equation: AUC_0‑*t*_/AUC_TR_ × 100, where “*t*” is time (min) and “TR” is the product’s label value.

The profiles were compared using a method based on the Similarity Factor (*F*2) according to eq 1. Dependent models were constructed using the definition of zero-order and first-order models, as a function of time (in minutes) versus the amount of undissolved ibuprofen. After 45 min of the dissolution test (Q_45_), the correlation coefficient (*r*), dissolution rate constant (*k*), dissolution half-life (*t*_50%_) (eq 2), and amount of dissolved ibuprofen were calculated.^18,19^

1 $$F2=50\;\log\left\{[1+\left(\frac{1}{n}\right)\sum_{t=1}^{n}{\left(\mathrm{Rt}-\mathrm{Tt}\right)}^{2}{]}^{-0.5}\times 100\right\}$$

2 $$T_{50\%}=0.693/k$$

### 2.7 HPLC-DAD Analysis and Analytical Validation

The ibuprofen reference standard (5 mg L^–1^) was injected (3.0 μL) using a mobile phase of ethanol (A) and 0.5% acetic acid (B) (v v^–1^) to identify and quantify ibuprofen using retention time and peak area. After optimizing the mobile phase, the following conditions were defined for ibuprofen analysis: The RP-C18 column was 4.6 × 100 mm with a particle size of 3.5 μm. The column temperature was 35 °C, and the flow rate was 0.8 mL min^–1^ for the solvent mixture of ethanol (70%, A) and acetic acid (0.5%, B). The chromatograms were acquired at 220 nm.

The chromatographic method was validated in accordance with official guidelines and the literature.^6^ The validation was based on the following criteria: linearity, limit of detection (LOD), limit of quantification (LOQ), selectivity (matrix effect), precision (intraday and interday), accuracy (addition and recovery), and robustness.^6,20–23^

The matrix effect was determined by comparing the angular coefficients of calibration curves constructed with ethanol and with the matrix (capsule excipients). Linearity was obtained through linear regression analysis of the correlation coefficient in the range of 1 to 20 mg L^–1^. Relative standard deviation (RSD, %) was used to calculate the quantification accuracy of the ibuprofen reference standard (5 mg L^–1^) on the same day and on three nonconsecutive days. Accuracy tests were performed using addition and recovery tests at three concentration levels (2, 5, and 10 mg L^–1^), in triplicate, and expressed as a percentage recovery of the analyte added to the sample. The detection and quantification limits were calculated using the method of Ribeiro et al.^21^ Robustness was evaluated through experimental design (2^2^), with variations in the mobile phase concentration (10% acetic acid) and column temperature at 1 °C.

### 2.8 Metrics for Assessing the Sustainability

Two complementary metrics were applied to assess the sustainability of the chromatographic analytical method: the National Environmental Methods Index (NEMI) diagram and the Analytical Greenness Metric (AGREE) tool. The NEMI diagram is a circular pictogram divided into four quadrants, each representing an environmental criterion: the use of toxic solvents or hazardous reagents, the generation of waste exceeding 50 g per analysis, the use of regulated reagents, and extreme pH (less than 2 or greater than 12). A green fill in each quadrant indicates compliance with the corresponding criterion, providing a simple visualization of the method’s environmental performance. However, this tool has two serious limitations: it does not consider the volumes of solvent and waste from the analytical procedure.^24–27^

On the other hand, the AGREE metric is based on the 12 principles of green analytical chemistry. It assigns a score from 0 to 1 to each principle, which is then weighted and processed using an algorithm. This generates a circular radial graph and a final numerical value between 0 (the least green) and 1 (the most green). Thus, while NEMI provides a quick qualitative analysis, AGREE enables a more comprehensive quantitative assessment of a method’s sustainability profile.^25–28^ The application available at https://mostwiedzy.pl/wojciech-wojnowski,174235–1/was used to evaluate the ″green profile″ for the AGREE method.^29^

### 2.9 Statistical Analysis

Statistical analyses were performed using Statistica software, version 7.0. A *p* value of less than 0.05 (95% confidence level) was considered significant. Analysis of variance (ANOVA) was used to analyze the experimental design results for the chromatographic and dissolution conditions.

## 3 Results and Discussion

### 3.1 General Tests (Weight Variation and Disintegration Tests)

Table 2 shows the results of the tests to determine weight uniformity, average weight, friability, and disintegration following the Brazilian Pharmacopoeia.^7^

**Table 2:** Physical Assay Results

	batch	average weight (mg ± DP)	disintegration time
	1494503	0.8349 ± 0.0228	10′34″
Reference	1530239	0.8499 ± 0.0265	07′24″
	1537996	0.8518 ± 0.0024	07′42″
	6029194	0.6511 ± 0.0165	06′50″
Similar	60280031	0.6607 ± 0.0102	06′10″
	60280037	0.6423 ± 0.092	06′50″
	241196	0.7030 ± 0.0296	06′13″
Generic	EKP12536	0.7007 ± 0.0128	07′01″
	EKP12578	0.6986 ± 0.0275	07′02″

According to the Brazilian Pharmacopoeia,^7^ the permitted variation for the average weight is ±7.5% (with an average weight above 300 mg). All nine samples met the test requirements. Compliance with these results suggests that the active ingredient is evenly distributed among the capsules and that the desired pharmacological effect will be achieved. In the disintegration test, none of the samples exceeded the 30 min time limit specified in the 2024 edition of the Brazilian Pharmacopoeia. This *in vitro* test measures the time it takes for the soft capsule to break down completely and release the active ingredient into the medium, allowing it to be dissolved and absorbed by the body to achieve the therapeutic effect.^30^

### 3.2 Analytical Validation of the Chromatographic Method

Table 3 presents the chromatographic validation parameters for the HPLC-DAD determinations of ibuprofen.

**Table 3:** Parameters of Chromatographic Validation for Ibuprofen by HPLC-DAD

parameter	result
Regression equation	*Y* = 8.795*x* – 0.598
Working range, in mg L^–1^	1.37–20
Correlation coefficient (*r*)	0.9997
Coefficient of determination (*R* ^2^)	0.9995
Matrix effect - regression equation^a^	*Y* = 8.811*x* – 0.837
Matrix effect - regression equation^b^	*Y* = 4.528*x* – 1.099
Limit of detection, in mg L^–1^	0.7768
Limit of quantification, in mg L^–1^	1.3673
Interday precision (RSD, %)	3.2
Intraday precision (RSD, %)	3.8
Average recovery (%)	
2.0 mg L^–1^	116.77 ± 9.01
5.0 mg L^–1^	123.74 ± 6.07
10.0 mg L^–1^	124.22 ± 9.06

a External analytical curve (ethanol).

b Sample addition analytical curve (matrix).

Analytical curves were generated to evaluate the matrix effect and were prepared in ethanol, as well as in the matrix medium (excipients in ethanol). The analytical curves (1.37 to 20.00 mg L^–1^), regression equations, and ratios of the analytical curves’ angular coefficients were obtained. The obtained ratio values indicated the matrix effect for ibuprofen; that is, the measured signal was influenced by interferents. However, the UV spectrum of the excipients does not absorb at the wavelength used to identify and quantify ibuprofen. Therefore, the excipients did not interfere with the detection or subsequent quantification of ibuprofen. A solution containing ibuprofen and 1% excipient was obtained. Its chromatogram confirmed that there was no spectral interference from the excipients in ibuprofen identification and determination, as shown in Figure 2. Thus, there was no overlap between the spectra of the excipients and those of ibuprofen, allowing for quantification without interference. Therefore, the calibration curve in ethanol can be used to quantify ibuprofen in the soft gelatin capsules.

**Figure 2:**
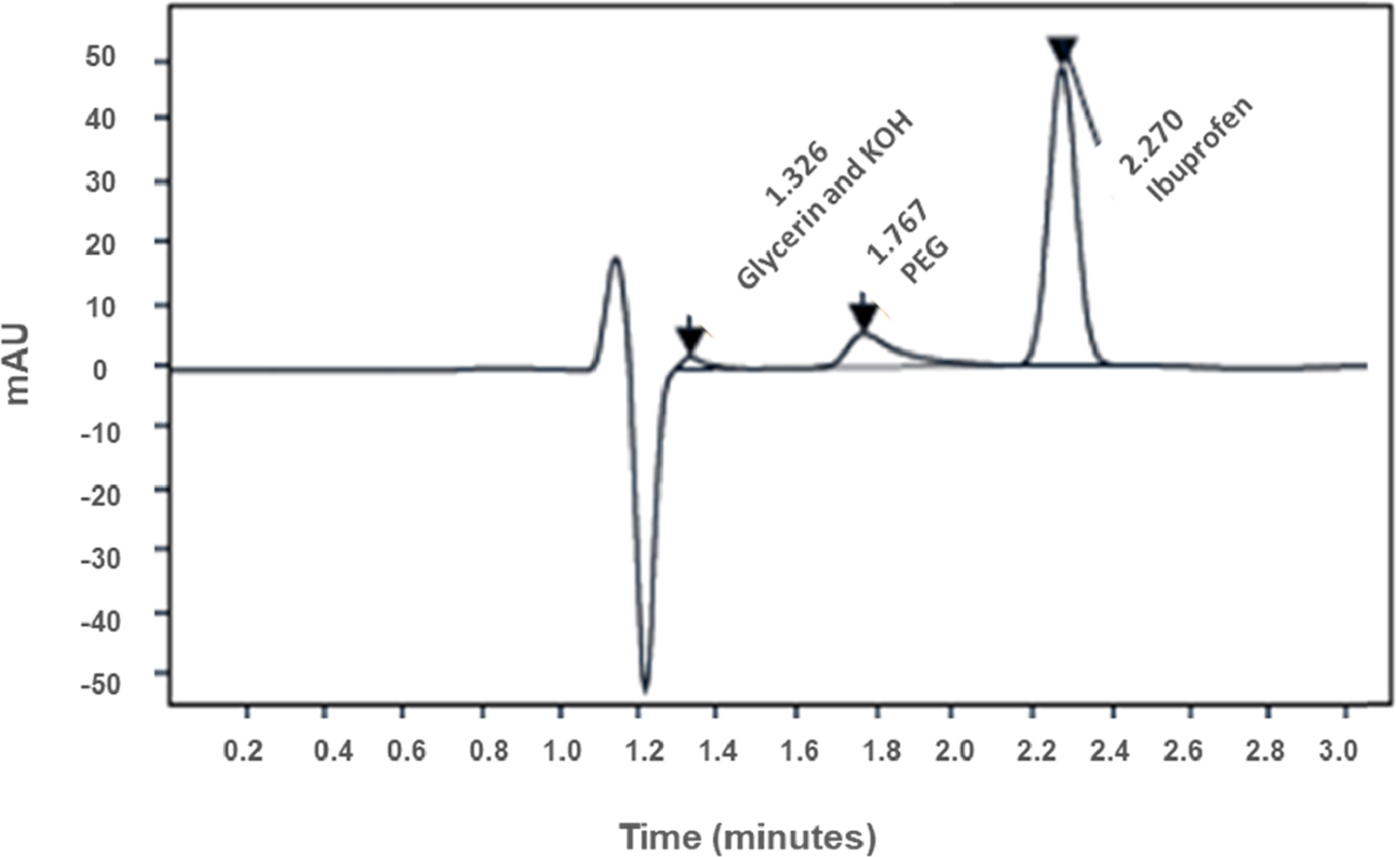
Chromatogram of 10 mg L^–1^ of Ibuprofen in a 1% solution of excipients in ethanol.

The LOD and LOQ were adequate for the detection and quantification of ibuprofen. Linearity was assessed based on the correlation coefficient (>0.99), which indicates a linear relationship between ibuprofen concentration and analytical signal. Precision was evaluated through 10 consecutive injections of the 5.0 mg L^–1^ standard ibuprofen solution (intra- and interday), with RSD values below 5%, which are within acceptable limits.^20,31^ Percentage recovery values above 80% were obtained at three concentration levels. Therefore, it can be inferred that the method produced adequate results for analytical validation. The method proved to be robust within the studied experimental domain (10% acetic acid concentration in the mobile phase at 0.5 ± 0.05% and a chromatographic column temperature of 1 °C). The Pareto chart (Figure 3) showed that the evaluated factors were not statistically significant at a 95% confidence level, demonstrating the robustness of the method.

**Figure 3:**
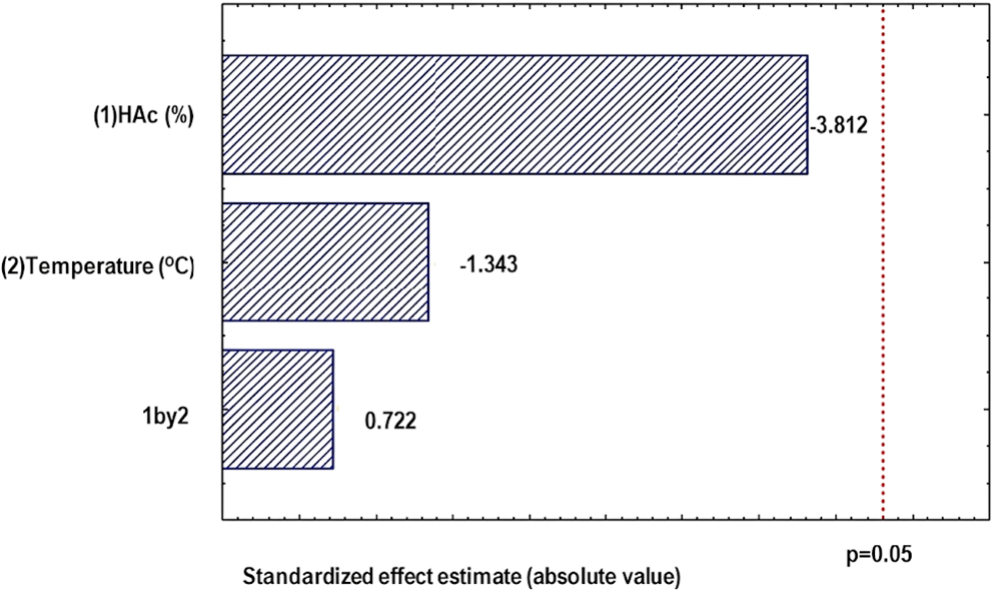
Pareto chart of the robustness of the chromatographic method.

### 3.3 Green Profile Assessment Metrics

NEMI is a simple green chemistry metric with a pictogram that represents a qualitative assessment of the analytical method.^25^ According to Figure 4(a), acetic acid is on the persistent, bioaccumulative, and toxic (PBT) list and ethanol is flammable. Therefore, the top two quadrants are blank, indicating that they are not sustainable according to the metrics. In the residue quadrant, the method involves a low residue (4 mL of solvent and 1.5 mL of sample). The last quadrant refers to pH; the mobile phase has a pH ranging from 2 to 12, which the NEMI method indicates as ideal (2/4). Figure 4(b) shows the AGREE calculation, which has a value of 0.71. The closer the value is to 1, the more sustainable the method is considered.^28^ Steps 7 and 10 are highlighted in yellow because the results were below the weighted average for the volume of solvent used per sample and the flammability risk associated with ethanol. Step 8 is highlighted in light green because our method only detects one analyte per run. However, we can analyze 12 samples per hour. Step 1 did not use the direct analytical technique; therefore, we need to use an offline analysis (HPLC-DAD), which is represented by the yellow color. In step 3, the analysis does not occur *in situ*; the HPLC works in offline mode.

**Figure 4:**
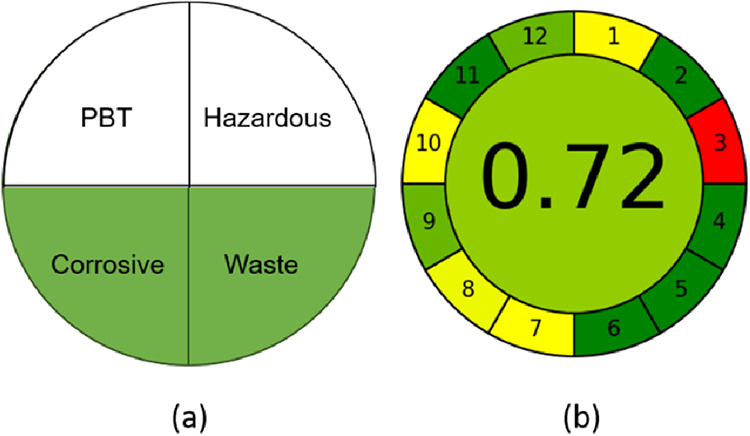
Assessment of the sustainability of the method developed using the NEMI (a) and AGREE (b) tools.

### 3.4 Factorial Experimental Design and Dissolution Test Conditions

A Pareto chart (Figure 5) was plotted based on the results of the dissolution tests with a 2^3^ factorial design and a triplicate of the central point to analyze the variables and their interactions. All three of the studied variables (volume, rpm, and time) were statistically significant (*p* > 0.05) independently. However, when evaluating the interactions, no interactions were statistically significant.

**Figure 5:**
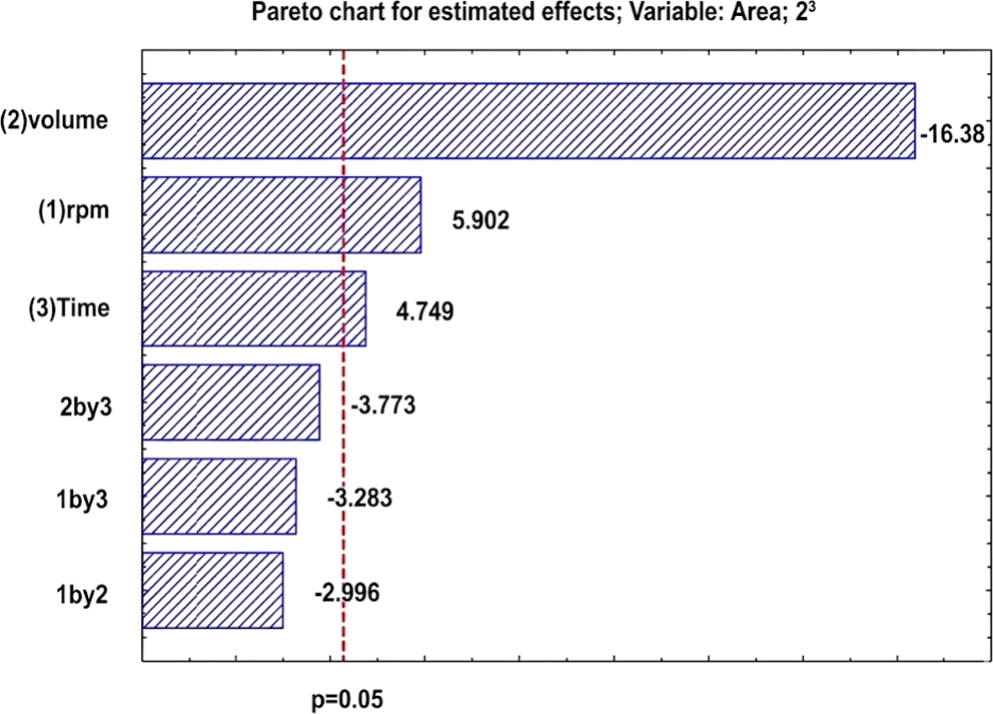
Pareto charts for the variables studied in dissolution.

The independent variables were optimized by using the response surface to maximize the system response. The Doehlert matrix was used because it is efficient and easy to apply to experimental variables, proving to be adequate and advantageous. Additionally, the levels of each variable can be arranged to provide more information about significant or problematic factors, allowing some factors to be studied with more or fewer levels.^32^ Thus, the three variables were maintained for optimization, as shown in Table 4.

**Table 4:** Doehlert Matrix for 3 Factors

experimental domain			
	rpm	Volume (mL)	Tempo (min)
Minimum	–1 (50)	–0.866 (300)	–0.817 (45)
Central point	0 (75)	0 (500)	0 (60)
Maximum	+1 (100)	0.866 (7000)	0.817 (75)
Experiment	rpm	Volume (mL)	Time (min)
1	0 (75)	0 (500)	0 (60)
2	1 (100)	0 (500)	0 (60)
3	0.5 (88)	0.866 (700)	0 (60)
4	0.5 (88)	0.289 (570)	0.817 (75)
5	–1 (50)	0 (500)	0 (60)
6	–0.5 (63)	–0.866 (300)	0 (60)
7	–0.5 (63)	–0.289 (450)	–0.817 (45)
8	0.5 (88)	–0.866 (300)	0 (60)
9	0.5 (88)	–0.289 (450)	–0.817 (45)
10	–0.5 (63)	0.866 (700)	0 (60)
11	0 (75)	0.577 (650)	–0.817 (45)
12	–0.5 (63)	0.289 (570)	0.817 (75)
13	0 (75)	–0.577 (370)	0.817 (75)
14	0 (75)	0 (500)	0 (60)
15	0 (75)	0 (500)	0 (60)
16	0 (75)	0 (500)	0 (60)

Response surface graphs were generated by using the Doehlert matrix (Figure 6) to evaluate curvature and assess the quadratic model.

**Figure 6:**
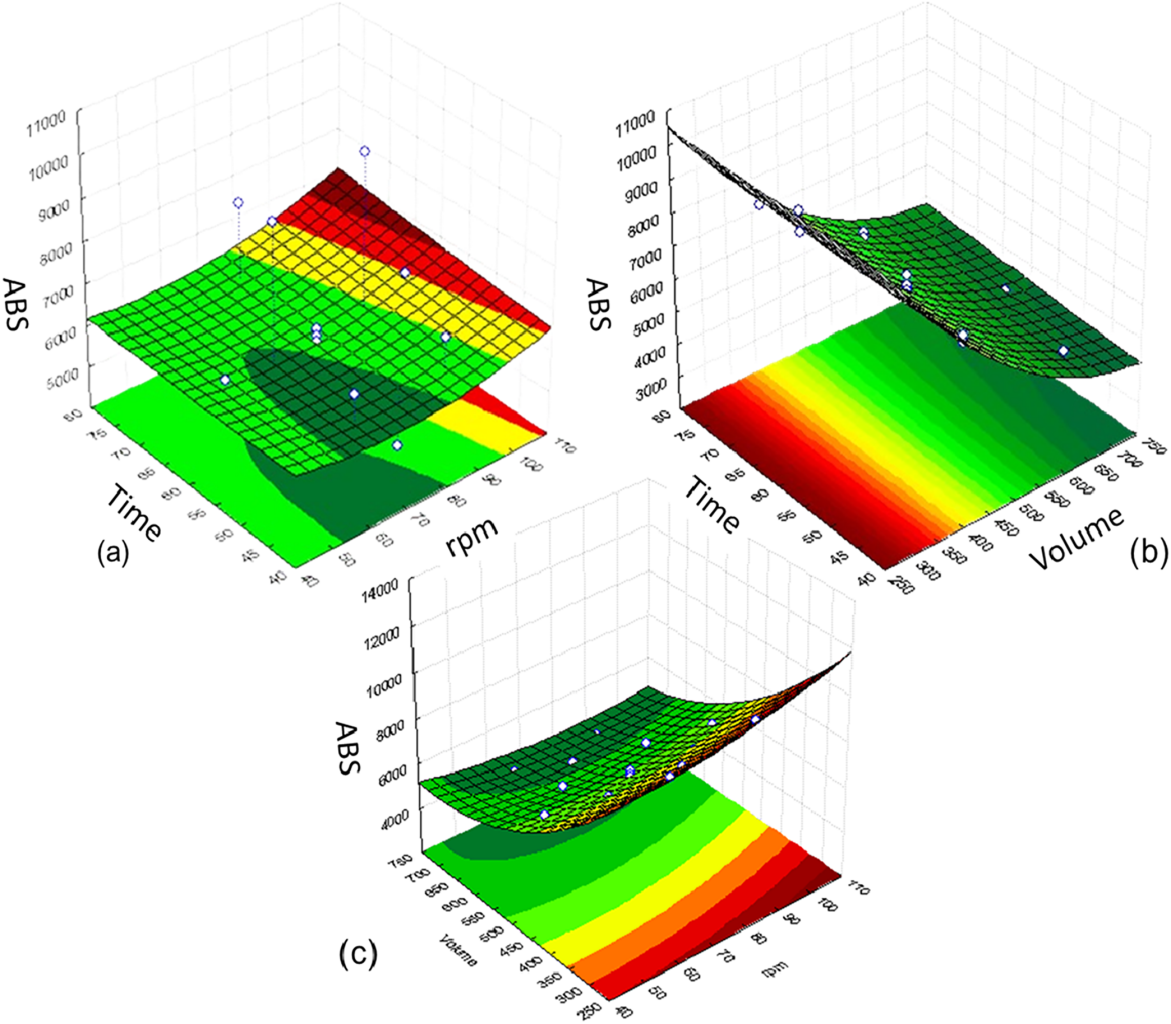
Response surface for (a) time × rpm; (b) time × volume; (c) volume × rpm; and mutual effects on absorbance (ABS).

The surfaces were relatively flat. Through ANOVA (Table 5), no significant differences were observed in the quadratic model for the rotation and time. This justifies the shape of the surface shown in Figure 6.

**Table 5:** Analysis of Variance (ANOVA)^a^

factor	SS	df	MS	F	p
**(1)** RPM (L)	229519	1	229519	13.256	0.035719
RPM (Q)	62294	1	62294	3.598	0.154115
**(2)** Volume (L)	31266756	1	31266756	1805.861	0.000029
Volume (Q)	1990767	1	1990767	114.980	0.001734
**(3)** Time (L)	52142	1	52142	3.012	0.181080
Time (Q)	749	1	749	0.043	0.848534
1L by 2L	115757	1	115757	6.686	0.081378
1L by 3L	4360	1	4360	0.252	0.650322
2L by 3L	10381	1	10381	0.600	0.495168
Lack of fit	79406	3	26469	1.529	0.367865
Pure error	51942	3	17314		
Total SS	33746343	15			

a SS: sum-of-squares; df: degrees of freedom; MS: mean square.

The critical values found were outside of the limits established in the experimental domain. Therefore, even if the domain is readjusted within the equipment conditions, the surface will likely remain linear. To define the volume of the medium, univariate planning was proposed with volumes ranging from 300 to 900 mL, increasing by 100 mL per experiment. The release percentages were then analyzed. Other conditions were maintained: The paddle was set to 75 rpm for 60 min, following the instructions in Anvisa’s Dissolution Guide applicable to the dissolution of immediate-release drugs for generic, new, and similar drugs.^16^

Since all volumes had a release rate of ≥80%, a *t-*test was applied to the 700 and 900 mL volumes because they had the highest release rates: 91.096% and 97.948%, respectively. However, the calculated value (0.01) was lower than the tabulated value (4.303). Therefore, the smallest volume of medium was used to save reagents. The optimal conditions were defined as follows: 700 mL of phosphate buffer at pH 7.2, 75 rpm, paddle, and 60 min.

### 3.5 Dissolution Studies

The dissolution process for immediate-release formulations generally takes 30 to 60 min. The dissolution profile of these products typically shows a gradual increase, reaching 85 to 100% within 30 to 45 min.^5^ With the newly developed method, eight of the nine samples reached 85% within 45 min, as shown in Figure 7. This figure is based on the dissolution profiles obtained for the pharmaceuticals studied.

**Figure 7:**
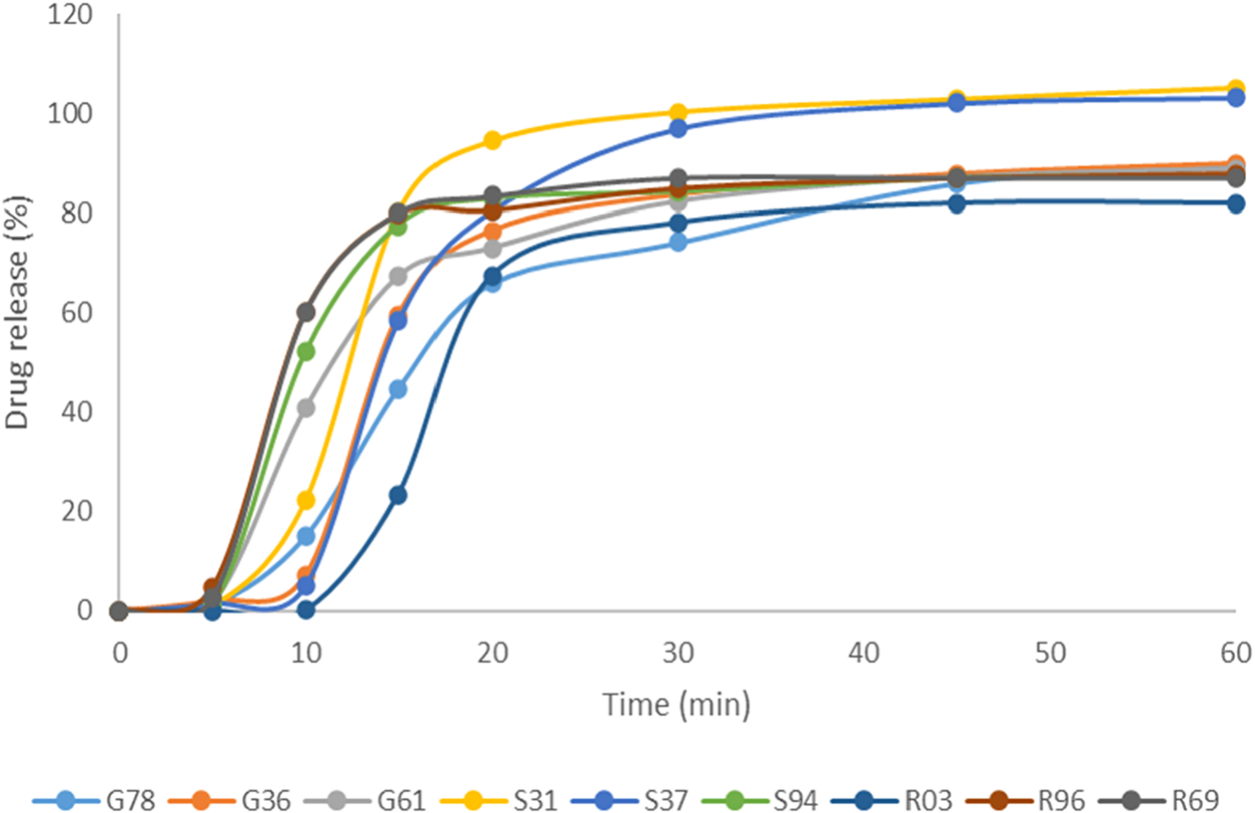
Comparison of Ibuprofen dissolution profiles (reference, similar, and generic) after optimizing the dissolution method.

Eraga et al.^15^ and Katona et al.^33^ conducted dissolution tests under the following conditions: 900 mL of phosphate buffer solution (pH 7.2), apparatus 2 (paddle), 50 rpm, and 60 min of testing.^33^ The amount of drug released in the Eraga et al.^15^ study varied from 39.6% to 101.5% after 60 min. Of the 10 tablets, only three showed a percentage above 85% released. At the end of the test, Katona et al.^33^ calculated that approximately 100% of the drug had been released from the soft capsule. Mayet-Cruz et al.^34^ used 900 mL of phosphate buffer (pH 7.2), apparatus 1 (basket), 150 rpm, and 45 min in their study. These conditions were applied to eight samples of ibuprofen soft capsules, all of which showed ≥ 90% release within 20 min.^34^

As shown in Figure 7, the R03 medication released only 81.96% of the drug at the end of the study compared to 87% for the other two batches. There was likely some deviation in the batch since, at the end of the dissolution time, the capsule had a bottom body, indicating that the drug remained attached to the casing. The other samples showed a release percentage ranging from 87.06% to 105.01%, demonstrating compliance with USP.^5^ However, as there is no monograph for ibuprofen soft capsules in the Brazilian Pharmacopoeia,^7^ there is no permitted content range as there is for tablets, which varies from 90% to 110% of the declared amount.^4,5^

As shown in Table 6, the dissolution efficiency (DE) ranged from 32.17% to 45.97%.

**Table 6:** Dissolution Efficiency (DE, %) of Ibuprofen Samples (Soft Gelatin Capsules)

samples	R03	R36	R96	G61	G36	G78	S31	S37	S94
**DE (%)**	32.17	42.23	42.76	39.19	37.38	34.31	45.97	41.73	41.58

In a Mexican study by Medina-López et al.,^35^ a method was proposed to quantify ibuprofen and caffeine simultaneously in soft gelatin capsules using UV–vis spectroscopy. The authors applied the USP dissolution method and achieved 99.05% release in 60 min, with ED at 61.39%. In a subsequent study, Abdelfattah et al.^36^ conducted dissolution testing on three brands of immediate-release ibuprofen (200 mg) tablets (one reference and two similar) to evaluate different pharmacopoeial apparatuses for dissolution testing. Following USP conditions, the drug release percentages ranged from 82.39% to 93.39%, with a DE (%) of 78.5% for the reference drug and 49.4% and 64.2% for the similar drugs.^36,37^ Both studies presented a DE (%) higher than that obtained in this study. However, Medina-López et al.^35^ evaluated an ibuprofen-caffeine association, while Abdelfattah et al.^36^ analyzed ibuprofen tablets. Thus, no equivalent studies on ibuprofen soft gelatin capsules could be found in the literature to compare with the results obtained in the present study.

Zero- and first-order kinetic models were calculated. The kinetic model selection was evaluated based on the correlation coefficient (r) closest to 1 because it best fits the dissolution profile of the evaluated drugs.^18,38^ In addition, the kinetic parameters obtained from the straight-line equations were determined by the first-order model: dissolution rate constant (k); dissolution half-life (t_50%_); and amount of ibuprofen dissolved in 45 min (Q45) (Table 7).

**Table 7:** Statistical Parameters of Regression Studies, Applying Zero-Order and First-Order Models, Derived from Dissolution Profiles

samples	zero-order	first-order	t_50%_	Q45 (%)	k (minutes ^‑1^)
R03	0.7591	0.8258	19.7436	81.958	0.0351
R69	0.5233	0.6472	20.1453	87.056	0.0344
R96	0.5434	0.7047	19.9712	86.873	0.0347
G61	0.6845	0.8729	17.7238	87.13	0.0391
G36	0.7081	0.8608	16.0789	87.704	0.0431
G78	0.8217	0.9561	16.7391	85.961	0.0414
S31	0.6646	0.8425	4.7047	102.754	0.1473
S37	0.7538	0.8919	5.7895	702.031	0.1197
S94	0.5619	0.7003	19.7436	86.82	0.0351

The correlation coefficients indicated that the first-order model produced better results for all nine samples. This model is characteristic of immediate-release drugs because it shows that the amount of drug released over time depends on the amount of drug remaining in the formulation.^39^

To compare the dissolution profiles, we calculated the similarity factor (*F*2) for each pair of profiles. According to Brazil,^40^ two profiles are considered equivalent when their *F*2 values fall within the range of 50 to 100. Table 8 shows the *F*2 values obtained for the studied drugs.

**Table 8:** *F*2 Values Resulting from Comparisons between Products

*F*2			
	R03	R69	R96
**G61**	32.06	48.43	**50.00**
**G36**	41.33	31.50	31.66
**G78**	48.96	30.10	30.57
**S31**	27.04	36.06	36.22
**S37**	35.67	25.55	29.76
**S94**	26.78	**69.20**	**70.27**

Among the samples studied, only three had results above expectations (*F*2 values between 50 and 100). Since this is a comparative study, if the reference drug in the test shows results below expectations, then its comparator will show results above normal. This is the case with R03, which showed only 81.96% when compared to 87% for the other two batches, as shown in Figure 7. When comparing the other batches with R03, no sample had *F*2 > 50, unlike batches R69 and R96, in which drug S94 showed a value within the similarity limits, and G61 when compared to R96.

No studies were found that performed *F*2 calculations for soft-capsule dosage forms. Abdelfattah et al.^36^ calculated the *F*2 value between the reference and similar ibuprofen-containing tablets; however, the values were also below the expected value. Katona et al.^33^ performed a Hungarian study in which they calculated *F*2 by comparing ibuprofen tablets with soft capsules (200 mg), which had an *F*2 value of 55. However, neither of these studies compared only ibuprofen soft gelatin capsules, making this study unique in the field.

These results indicate that batches R69, R96, and S94 are pharmaceutically equivalent.^7^ This means they can be interchanged at the time of dispensing because it is assumed that they will have equivalent efficacy, safety, and potential to cause adverse effects when administered to the body.

## 4 Conclusion

Due to the recent commercialization of ibuprofen soft gelatin capsules, studying the quality control of this pharmaceutical form became necessary. The weight uniformity and disintegration showed compliance with the requirements of the Brazilian Pharmacopeia. A more sustainable method was developed by replacing acetonitrile or methanol (widely used in literature) with ethanol. The method was validated according to guidelines (ICH and Anvisa) and applied for ibuprofen soft gelatin capsules assay. To determine ibuprofen by HPLC-DAD were an isocratic mobile phase consisting of 70% ethanol and 30% of 0.5% acetic acid at a flow rate of 0.8 mL min^–1^, a column temperature of 35 °C, wavelength of 220 nm, a total running time of 5 min. There are no methodologies for testing dissolution, in the Brazilian Pharmacopeia, of a soft capsule containing ibuprofen. The application of factorial design 2^3^ allowed to investigated factors for the development of a dissolution test, subsequently optimized using the Doehlert response surface matrix. The dissolution conditions were defined as follows: 700 mL of phosphate buffer solution at pH 7.2; apparatus 2 (paddle); 75 rpm; and 60 min. The DE values ranged from 32.17% to 45.97%. As no other studies were found in the literature that calculated DE using only ibuprofen soft gelatin capsules, comparisons could not be made. The drug showed a first-order dissolution kinetics model based on determining the dissolution rate constant, dissolution half-life, and amount of ibuprofen dissolved in 45 min. In the absence of studies and monographs on the dissolution of ibuprofen soft gelatin capsules, this study contributes to the Brazilian Pharmacopoeia and to worldwide research on quality control and sustainable method development.

## §

Department of Life Sciences, Postgraduate Program in Pharmaceutical Sciences (PPGFARMA), State University of Bahia (UNEB), Silveira Martins, 2555, Cabula, Salvador 41150–000, Bahia, Brazil
